# Impact of Checklist Implementation on Endotracheal Intubation‐Related Complications in Critically Ill Adults—A Retrospective Before and After Study

**DOI:** 10.1111/aas.70103

**Published:** 2025-07-22

**Authors:** Atosa Nejatian, Ida‐Maria Forsberg, Ulrika Thorö, Albin Sjöblom, Jessica Kåhlin

**Affiliations:** ^1^ Perioperative Medicine and Intensive Care Karolinska University Hospital Stockholm Sweden; ^2^ Department of Physiology and Pharmacology, Section for Anaesthesiology and Intensive Care Medicine Karolinska Institutet Stockholm Sweden

**Keywords:** adult, airway management, checklist, critical care, critical illness, hypotension, hypoxia, intensive care unit, intubation, intubation complications

## Abstract

**Background:**

Endotracheal intubation is a common procedure in the intensive care. However, it is associated with a high incidence of complications, primarily hypotension and hypoxia. Although guidelines advocate the use of checklists for intubation, studies on their effectiveness are limited. The aim was to investigate whether the implementation of a checklist for intubation in an intensive care unit is associated with a reduction in immediate intubation‐related complications.

**Materials and Methods:**

We conducted an observational before and after study, implementing an intubation checklist before the second period. Adult patients intubated between December 2022 and August 2024 at Karolinska University Hospital were included. Data regarding intubations were collected from a quality registry. The primary outcome was a SpO_2_ < 90% by pulse oximetry or systolic arterial pressure (SAP) < 90 mmHg within 5 min of intubation. Chi‐square or Fisher's exact test was used for the comparison of proportions.

**Results:**

A total of 97 patients were included. There was no significant difference in the primary outcomes of SpO_2_ < 90% (30% vs. 21%, *p* = 0.29) or SAP < 90 mmHg (34% vs. 32%, *p* = 0.80) before and after checklist implementation. There was no difference in the secondary outcomes of lowest median SpO_2_ from intubation until up to 5 min after, surgical airway intervention, cardiovascular collapse, cardiac arrest, oesophageal intubation, severe arrhythmia, or death between the periods. Apnoeic oxygenation, either by standard nasal cannula or high‐flow nasal cannula, was used more frequently after checklist implementation (7% vs. 36%, *p* = 0.005). Preoxygenation with non‐invasive ventilation was used in 33% of cases in the standard period compared to 48% in the checklist period (*p* = 0.19).

**Conclusions:**

In this study on implementing a checklist for intubation of critically ill adults, we could not demonstrate a reduction in immediate intubation‐related complications. However, the use of apnoeic oxygenation increased in the checklist period, highlighting the checklist's value as a cognitive aid when preparing for this common yet perilous procedure.

**Editorial Comment:**

This time interrupted series analysis looked at implementation of a checklist for preparing and planning intubation of ICU patients, where it is recognized that there is risk for respiratory or circulatory adverse events. The findings here from one center showed that implementation of new, detailed checklist‐based preparation can affect practice, and that when advanced non‐invasive positive pressure ventilatory support is routinely applied, hypoxia can be less frequent then before these were routinely implemented.

**Trial Registration:** Clinicaltrials.gov identifier: (NCT06791317)

**Abbreviations**
CRF: case report form
ETI: endotracheal intubation
HFNC: high‐flow nasal cannula
ICU: intensive care unit
NAP4: The UK 4th National Audit Project
NIV: non‐invasive ventilation
SAP: systolic arterial pressure
SpO_2_
: peripheral arterial oxygen saturation

## 1 Introduction

Endotracheal intubation (ETI) is among the most frequently performed procedures in the intensive care unit (ICU), where effective and timely airway management is critical to avoid complications [1]. However, it is associated with a high incidence of adverse events, as studies indicate that approximately 45% of cases experience immediate intubation‐related complications, primarily hypotension and hypoxia [2, 3]. Cardiac arrest occurs in approximately 3% of intubations [2, 4]. Critically ill patients often present with physiologically challenging airways due to unstable respiratory or hemodynamic states, significantly increasing the risk of complications. Furthermore, they have limited physiological reserve, presenting unique challenges for intensivists [1, 3]. Factors that contribute to higher complication rates are insufficient preoxygenation and planning, inexperienced staff, and inadequate setting and equipment [4, 5]. Additionally, hypotension, hypoxia, and the absence of preoxygenation before ETI are associated with an increased risk of cardiac arrest [2, 4]. These findings suggest that, while ETI is a common procedure, it presents significant risks for patients.

In response to these risks, guidelines advocate the use of structured checklists for intubation in the ICU [1, 5, 6]. Checklists serve as cognitive and practical aids to systematically plan and optimise procedures, thereby enhancing adherence to established protocols. Checklists promote standardisation, which reduces reliance on individual memory, which is often compromised by stress and fatigue [7]. Additionally, previous studies indicate that checklists foster collaborative environments, ensuring that essential components are not omitted [8, 9]. Thus, ETI is an ideal candidate for the implementation of a checklist approach in critically ill patients in the ICU.

In the intensive care setting, bundle management protocols for intubation have been associated with a reduction in immediate life‐threatening complications, such as severe hypoxia and cardiovascular collapse [10], and an increase in first‐pass success [11]. In contrast, a randomised controlled trial found no differences in intubation‐related complications when using a checklist for intubation, compared to not using a checklist [12]. Few studies have examined intubation checklists in adult ICUs, and existing research presents contradictory findings [10, 11, 12, 13], making checklist design and implementation challenging.

Therefore, we aimed to investigate whether the implementation of a pre‐procedural checklist for intubation in the ICU could reduce intubation‐related complications, such as hypoxia and hypotension, in critically ill patients.

## 2 Methods

### 2.1 Aim

Our aim was to study whether the introduction and implementation of a pre‐procedural checklist for intubation in the adult ICU is associated with a decreased incidence of immediate intubation‐related complications. We hypothesised that the checklist could reduce the occurrence of hypoxic and hypotensive events.

### 2.2 Study Design

The study had a retrospective observational two‐phase design. A quality registry of ICU intubations was introduced in the four‐month standard period, where intubation procedures were performed according to routine care. An implementation period of 5 months followed, where the checklist was introduced. During the subsequent 10‐month checklist period, intubations were performed using the checklist. Except for the checklist, all other aspects of the ETI procedure were left to the discretion of the responsible consultant in anaesthesia and intensive care. The physicians performing the intubations were residents and consultants in anaesthesia and intensive care.

The study was conducted in two ICUs at the Karolinska University Hospital, Stockholm, between December 2022 and August 2024. The study was approved by the Swedish ethical review authority (Dnr 2023‐03310‐01) with a waiver of informed consent.

### 2.3 Study Population

Adult patients, 18 years and older, undergoing ETI for any indication in the ICU were eligible for inclusion. Patients were excluded if information regarding their intubation had not been registered in the database due to missing completed case report forms (CRF) for either period, or if the checklist had not been used for any reason during the checklist period.

### 2.4 The Checklist

Before the checklist period, a checklist was compiled by an appointed task force in our department (Appendix A). The checklist was developed based on current ICU airway guidelines and relevant literature [6, 10, 14, 15, 16, 17, 18] through an iterative process of refinement within the group, prior to adoption. It was then introduced to anaesthetists, residents, nurses, and assistant nurses working at the ICUs. During and after the implementation, it was also used in team trainings and airway simulations. The checklist was organised into categories of physiological optimisation, equipment and drugs, team line‐up, airway, and contingency plan.

### 2.5 Measurements and Outcomes

Data regarding the intubations were collected from the quality registry at the ICU. The quality registry contains information on ICU intubations, including parameters such as pulse, blood pressure, and saturation before, during, and up to 5 min after intubation. These parameters, complications, and other intubation‐related information are manually registered on a CRF, bedside, and entered into a database. A second person present during the intubation recorded complications on the same CRF. Data on demographics, reasons for admission, and follow‐up data were collected from patient charts.

The primary outcome was defined as the occurrence of any of the following: (I) a peripheral arterial oxygen saturation (SpO_2_) below 90% or a five‐percentage point reduction or more if SpO_2_ was < 90% following preoxygenation as measured by pulse oximetry, or (II) a systolic arterial pressure (SAP) below 90 mmHg, from intubation up to 5 min after the procedure.

Secondary outcomes included the lowest median SpO_2_ from intubation until up to 5 min after, immediate life‐threatening complications such as surgical airway intervention, cardiovascular collapse, cardiac arrest, oesophageal intubation, severe arrhythmia, and death. Additional metrics included any change of method or operator, duration of intubation (i.e., time from laryngoscopy to end‐tidal carbon dioxide measurement), perceived intubation difficulty, the number of intubation attempts, 30‐day mortality, length of ICU stay, and ventilator days.

Exploratory post hoc analyses included SpO_2_ < 80% and 70%, SAP below 80 and 70 mmHg, and the lowest median SAP, as well as the use of a device for preoxygenation, apnoeic oxygenation, the urgency of intubation, airway management, and the intubation procedure. In addition, exploratory post hoc subgroup analyses were performed on patients with respiratory failure intubated with immediate urgency.

### 2.6 Statistics

Data were expressed as median and interquartile range, or number and percentage. Proportions were compared using the chi‐square test or Fisher's exact test, as appropriate. Comparisons of medians were analysed with the Mann–Whitney *U* test. Logistic regression was used to adjust for confounding factors. A *p*‐value of < 0.05 was considered statistically significant. All data were compared between the standard and checklist periods. Analyses were conducted using SPSS version 29.0.

## 3 Results

**FIGURE 1:**
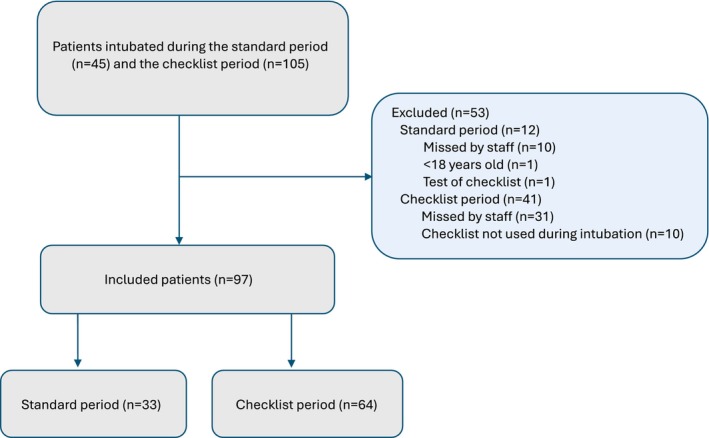
Patient inclusion. Critically ill patients intubated in two ICUs were included between December 2022 and April 2023 (standard period) and September 2023–August 2024 (checklist period) at Karolinska University Hospital, Stockholm, Sweden.

During the study period, 150 critically ill patients required intubation in the two ICUs studied. A total of 97 patients were included in the study (Figure 1), 33 patients in the standard period and 64 patients in the checklist period. Baseline characteristics were similar for both groups (Table 1) with a median age of 63 (49–72) years and 42% females in the full cohort.

**TABLE 1:** Baseline characteristics.

	Standard period (*n* = 33)	Checklist period (*n* = 64)
Patient characteristics		
Age (years)	68 (54–74)	61 (46–72)
Female sex	13 (39%)	28 (44%)
SAPS III score^a^	65 (61–79)	64 (52–77)
BMI (kg/m^2^)	22 (20–27)	26 (22–29)
GCS score before preoxygenation^a^		
13–15	12 (38%)	29 (48%)
9–12	12 (38%)	12 (20%)
3–8	8 (25%)	19 (32%)
Reason for ICU admission		
Neurological	16 (49%)	28 (44%)
Respiratory failure	13 (39%)	26 (41%)
Sepsis/Infection	6 (18%)	12 (19%)
Trauma	2 (6%)	5 (8%)
Airway	1 (3%)	4 (6%)
Circulatory failure	1 (3%)	2 (3%)
Other	2 (6%)	2 (3%)
Reason for intubation		
Respiratory failure	24 (73%)	41 (64%)
Altered conscioussness	14 (42%)	21 (33%)
Airway	6 (18%)	7 (11%)
Delirium	5 (15%)	6 (9%)
Intervention	4 (12%)	4 (6%)
Circulatory failure	4 (12%)	2 (3%)
Other	4 (12%)	9 (14%)
Co‐morbidities		
Respiratory disease	15 (46%)	19 (30%)
Hypertension^a^	14 (42%)	26 (41%)
Cancer^a^	9 (29%)	15 (23%)
Heart failure	7 (21%)	16 (25%)
Atrial fibrillation^a^	6 (21%)	14 (22%)
Diabetes^a^	6 (18%)	11 (17%)
Previous myocardial infarct^a^	1 (3%)	4 (6%)
Pulmonary embolism^b^	6 (18%)	1 (2%)

*Note:* Data are number (%) or median (IQR). One patient can have more than one reason for ICU admission or for intubation.

Abbreviations: BMI, body mass index; GCS, glasgow coma scale; ICU, intensive care unit; SAPS III, simplified acute physiologic score.

^a^ Missing data in up to five patients.

^b^ Pulmonary embolism was found during the hospital stay before the time of intubation.

### 3.1 Primary Outcomes

There was no difference in the primary outcomes SpO_2_< 90% or SAP < 90 mmHg before and after checklist implementation (Table 2). Desaturation below 90% occurred in 30% of patients during the standard period and 21% during the checklist period (*p* = 0.29). Hypotension, defined as SAP below 90 mmHg, occurred in 34% of patients before implementation and in 32% after implementation of the checklist (*p* = 0.80).

**TABLE 2:** Primary and secondary outcomes.

	Standard period (*n* = 33)	Checklist period (*n* = 64)	*p*
Any complication^a^	17 (52%)	29 (46%)	0.61
Primary outcome			
SpO_2_ < 90%^a^	10 (30%)	13 (21%)	0.29
SAP < 90 mmHg^a^	11 (34%)	20 (32%)	0.80
Secondary outcomes			
Lowest median SpO_2_ ^a^ (%)	95 (88–99)	97 (90–100)	0.33
Emergency surgical airway	1 (3%)	0 (0%)	0.34
Cardiovascular collapse^b^	1 (3%)	5 (8%)	0.66
Cardiac arrest	0 (0%)	0 (0%)	
Oesophageal intubation	0 (0%)	0 (0%)	
Severe arrhythmia	0 (0%)	0 (0%)	
Death (procedural)	0 (0%)	0 (0%)	
Change of method/operator	3 (9%)	5 (8%)	1.00
Duration of intubation (s)^c^	83 (48–124)	69 (48–104)	0.32
Intubation difficulty^a^			
Easy	24 (73%)	51 (81%)	0.35
Complicated	8 (24%)	12 (19%)	0.55
Impossible	1 (3%)	0 (0%)	0.34
Number of intubation attempts^a^			
1	27 (90%)	55 (86%)	0.75
2	3 (10%)	9 (14%)	0.48
30‐day mortality^d^	14 (42%)	14 (22%)	0.03
Length of ICU stay (days)	6 (3–12)	10 (6–16)	0.02
Length of mechanical ventilation (days)^a^	5 (3–10)	8 (5–14)	0.03
Other outcomes^a^			
SpO_2_ < 80%	4 (13%)	5 (8%)	0.48
SpO_2_ < 70%	1 (3%)	3 (5%)	1.00
SAP < 80 mmHg	6 (19%)	14 (22%)	0.70
SAP < 70 mmHg	5 (16%)	9 (14%)	1.00
Lowest median SAP^a^ (mmHg)	103 (87–119)	110 (88–130)	0.32

*Note:* Data are number (%) or median (IQR). Lowest median SpO_2_ and SAP are from intubation up to 5 min after. Duration of intubation is defined as time from laryngoscopy to end‐tidal carbon dioxide measurement. SpO_2_, peripheral arterial oxygen saturation, SAP, systolic arterial pressure. A *p* < 0.05 was considered significant.

^a^ Missing data in up to three patients.

^b^ Defined as SAP < 65 mmHg.

^c^ Missing data for 20 patients.

^d^ The significance for 30‐day mortality disappeared when adjusted for co‐morbidities, age, pulmonary embolism, previous smoking, and SAPS‐score.

### 3.2 Secondary Outcomes

**FIGURE 2:**
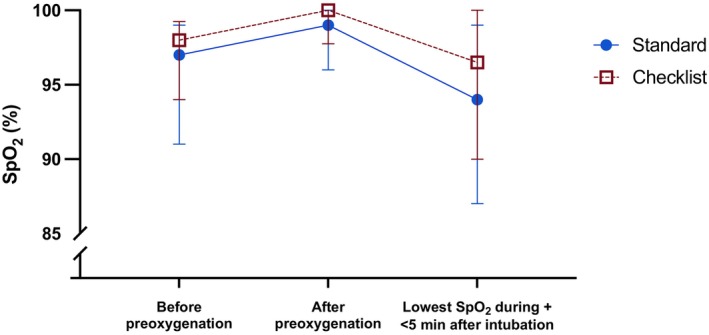
Lowest median SpO_2_. The lowest median SpO_2_ and interquartile range at different time points (before and after preoxygenation, during and up to 5 min after intubation) in critically ill patients intubated in the ICU in the standard period and in the checklist period. No difference between the groups was seen.

There were no differences in the secondary outcomes of lowest median SpO_2_ from intubation until up to 5 min thereafter, surgical airway intervention, cardiovascular collapse, cardiac arrest, oesophageal intubation, severe arrhythmia, or death between the periods (Table 2, Figure 2). Similarly, there were no differences in change of method, duration of intubation, intubation difficulty, or the number of intubation attempts between the standard and checklist periods (Table 2). A lower 30‐day mortality rate (42% vs. 22%, *p* = 0.03) was observed in the checklist period, which was not seen when adjusted for age, SAPS, pulmonary embolism, the comorbidities respiratory disease and atrial fibrillation, and previous smoking. A longer ICU stay (6 [3, 4, 5, 6, 7, 8, 9, 10, 11, 12] vs. 9.5 [6, 7, 8, 9, 10, 11, 12, 13, 14, 15, 16] days, *p* = 0.02) and length of mechanical ventilation (5 [3, 4, 5, 6, 7, 8, 9, 10] vs. 8 [5, 6, 7, 8, 9, 10, 11, 12, 13, 14] days, *p* = 0.03) were observed in the checklist period (Table 2).

### 3.3 Intubation and Airway Management

Intubations were performed by anaesthetists or residents in anaesthesia; the two phases did not differ in operator experience (Table 3). Intubations in the standard period were more often performed during on‐call hours (76% vs. 55%, *p* = 0.04). Apnoeic oxygenation, either by standard nasal cannula or high‐flow nasal cannula (HFNC) was performed more frequently in the checklist period (7% vs. 36%, *p* = 0.005). Preoxygenation with non‐invasive ventilation (NIV) was used in 33% of cases in the standard period compared to 48% in the checklist period (*p* = 0.185), 35% of the patients in the checklist period vs. 45% of the patients in the standard period had NIV at the time of the intubation decision. Video laryngoscopy was used in 94% versus 97% (*p* = 0.60), and an intubation stylet was used in 50% versus 63% (*p* = 0.22) in the standard versus checklist period, respectively (Table 3).

**TABLE 3:** Airway management.

	Standard period (*n* = 33)	Checklist period (*n* = 64)	*p*
Intubated on ICU day			
1st day	26 (79%)	36 (56%)	0.029
2nd day	1 (3%)	8 (13%)	0.16
3rd day	2 (6%)	6 (9%)	0.71
Other	4 (12%)	14 (22%)	0.24
Preoxygenation procedure			
Tight facial mask^a^	16 (59%)	26 (41%)	0.10
NIV^a^	9 (33%)	31 (48%)	0.19
HFNC^a^	1 (4%)	9 (14%)	0.27
Other^a^	2 (7%)	1 (2%)	0.21
Intubation procedure			
On‐call hours intubation^b^	25 (76%)	35 (55%)	0.04
Mask ventilation during preoxygenation^c^	5 (23%)	7 (12%)	0.29
Apnoeic oxygenation^a^	2 (7%)	23 (36%)	0.005
Standard nasal cannula	1 (4%)	14 (22%)	0.03
HFNC	1 (4%)	9 (14%)	0.27
Video laryngoscopy^a^	30 (94%)	62 (97%)	0.60
Intubation stylet^a^	16 (50%)	38 (63%)	0.22
Operator experience > 5 years	29 (88%)	48 (75%)	0.14
Oxygen support before intubation decision	31 (94%)	62 (97%)	0.60
Facial mask^a^	12 (39%)	10 (16%)	0.02
HFNC^a^	4 (13%)	8 (13%)	1.00
NIV^a^	11 (35%)	28 (45%)	0.20
Other	1 (3%)	1 (2%)	1.00
Unknown method^a^	3 (10%)	15 (24%)	0.10
Emergency of intubation^a^			
Immediate emergency	8 (27%)	16 (25%)	0.90
Emergency (< 1 h)	17 (57%)	37 (59%)	0.85
> 1 h	5 (17%)	10 (16%)	1.00

*Note:* Data are number (%) or median (IQR). NIV, non‐invasive ventilation; HFNC, high‐flow nasal cannula. A *p* < 0.05 was considered significant.

^a^ Missing data in up to seven patients.

^b^ On‐call hours are defined as weekdays 4 p.m. to 7.30 a.m. or weekends (friday 2.30 p.m. to monday 7.30 a.m.).

^c^ Missing data for 16 patients.

### 3.4 Exploratory Post Hoc Subgroup Analyses

Post hoc subgroup analyses were performed on the patient group that was intubated due to respiratory failure, with immediate urgency (*n* = 15). A lower number of patients desaturated below 90% (14% vs. 86%, *p* = 0.03) in the checklist period compared to the standard period, and the median SpO_2_ was lower during and up to 5 min after intubation in the standard period compared to the checklist period, *p* = 0.03 (Table B1).

Further subgroup analyses of patients that were intubated due to respiratory failure, with any emergence of intubation (*n* = 64) revealed that a higher proportion of patients had received apnoeic oxygenation (9% vs. 37%, *p* = 0.015) and were more commonly preoxygenated with NIV (35% vs. 76%, *p* = 0.001) after checklist implementation. NIV was more commonly used in this group before preoxygenation (43% vs. 72%, *p* = 0.03).

## 4 Discussion

In the present study on implementing a checklist for intubation of critically ill adults in the ICU, we did not demonstrate a reduction in immediate intubation‐related complications, primarily defined as hypoxia (SpO_2_ < 90%) or hypotension (SAP < 90 mmHg).

Although the primary outcome of hypoxia was not statistically significant, we noted that 30% versus 21% of the patients desaturated below 90% before and after checklist implementation. The lack of statistical significance may be attributed to our sample size, with only 33 patients in the standard period. In contrast to previous studies that considered only severe hypoxia, SpO_2_ < 80%, our study design also considered SpO_2_ < 90% [2, 3, 4, 10, 11]. Further, our study did not observe a reduction in SAP below 90 mmHg. This study considered even a momentary blood pressure below 90 mmHg compared to other studies that considered either a longer duration of hypotension or a lower threshold [2, 3, 4, 10, 11]. This strict approach could contribute to the lack of improvement in hypotensive episodes. While hypotensive events in our study are frequent, previous studies have shown higher rates following intubation [2]. Other studies measured complications up to 1 h after intubation [2, 3, 4, 10, 11], while our study focused on immediate intubation‐related complications within 5 min post‐procedure, as more extended observation periods could complicate claims of causality for adverse events.

A lower 30‐day mortality rate was observed after checklist implementation, and a longer length of ICU stay and length of mechanical ventilation. We do not believe there is a causality between 30‐day mortality and the checklist implementation as there could be several reasons for these outcomes. The difference disappeared when adjusted for co‐morbidities, age, pulmonary embolism, previous smoking, and SAPS‐score. The difference in length of ICU stay and length of mechanical ventilation could either reflect a sicker population that needs the treatment for a longer time, or reflect a higher mortality and thus a shorter treatment time.

In contrast to a previous study improving first‐pass success for intubation [11], our department had already a very high first‐pass success rate (86%) before checklist implementation. This could likely be explained by the presence of experienced operators, regular multidisciplinary team trainings on airway management, the Scandinavian anaesthesia and intensive care system where residency includes both anaesthesia and intensive care and thus gives each operator more routine in airway management, and the frequent use of video laryngoscopy (94%) [17, 19].

Previous studies have shown the benefit of apnoeic oxygenation, either by standard nasal cannula or by HFNC, as it prolongs the safe apnoeic time and time to desaturation [20, 21, 22, 23], a standard nasal cannula is recommended at our ICU, as stated in the checklist. In the checklist period, we noted an increased use of apnoeic oxygenation. NIV used for preoxygenation is recognised for its ability to reduce hypoxia in patients with respiratory failure undergoing ETI [24]. The recent randomised PREOXI trial demonstrated a reduction in hypoxia by approximately half in critically ill patients undergoing emergency ETI when preoxygenated with NIV [25]. This study demonstrated a trend towards increased use of NIV for preoxygenation following checklist implementation, and higher use in specific subgroups. However, the increase in preoxygenation with NIV after checklist implementation is attributable mainly to NIV dependency before airway management.

Before checklist implementation, our ICU had already maintained high standards regarding preoxygenation, the use of capnography, video laryngoscopy, and the presence of multiple clinicians for intubations. The checklist functioned primarily as a reminder to tailor the procedure to the patient's needs, leaving all aspects of intubation at the discretion of the anaesthetist in charge. This approach differs from those of Jaber et al. and Corl et al., who used protocols that included specific interventions to which clinicians were expected to adhere [10, 11]. Unlike the checklist employed by Janz et al., which included only the most commonly used items, our version also reminded clinicians to optimise physiological parameters, such as hemodynamic and neurological care in addition to airway management [12].

Patients intubated for respiratory failure with immediate urgency desaturated below 90% to a lesser extent in the checklist period, and the lowest median SpO_2_ from intubation up to 5 min after was higher. However, a conclusion from these results is limited by the small sample size. Nevertheless, in this high‐risk group of patients, time to desaturation is shorter, and the procedure is urgent and stressful. In this challenging setting, a structured checklist may reduce complications related to immediate airway management.

This study has several limitations. Firstly, the two‐phase retrospective observational design does not sufficiently establish causality; however, baseline characteristics were similar for both groups. The study design may have underestimated the actual effect of the checklist but also minimised the risk of significant cross‐contamination by participants becoming overly familiar with the checklist. Secondly, we cannot report full adherence to the checklist for every intubation during the study period. Thirdly, there may be reporting bias because clinicians partly self‐reported complications. However, a second person recorded complications on the same CRF, and study group members were randomly present for some procedures. Given that complication rates in our study were similar to previous studies, this is not a major concern. Fourthly, blood pressure was recorded only from the start of laryngoscopy, not immediately after induction, so the true blood pressure nadir may have been missed. This specific data is unfortunately not available in the quality registry. Fifthly, the study size, particularly in the standard period, may have been insufficient for detecting statistical significance. Unfortunately, the database with intubation‐related information was created shortly before the decision to implement the checklist.

While the implementation of a checklist did not reduce immediate intubation‐related complications such as hypoxia or hypotension in this study, we noted an increased use of apnoeic oxygenation with standard nasal cannula in the checklist period. This highlights the checklist's potential to enhance adherence to best practices and ensure safe intubation procedures. Applying the checklist to a variety of critically ill adult patients requiring intubation in our ICUs supports the generalisability of our results. We believe that a straightforward intervention, such as an intubation checklist, can be easily implemented and provide benefits and cognitive support in both ICUs and facilities with infrequent intubation procedures. However, its efficacy may be influenced by different settings and subgroups of patients, the specific contents of the checklist, and operator experience. Although this study was conducted in a highly resourced ICU with a frequent use of video laryngoscopy, we believe that an intubation checklist may prove particularly beneficial for inexperienced operators or in resource‐limited environments by adding structure, a contingency plan, and cognitive aid. Further and larger trials are needed in different settings to establish the definitive role and effectiveness of intubation checklists, hoping to improve patient outcomes.

## 5 Conclusion

In this study on implementing an intubation checklist for critically ill adults in the ICU, we could not demonstrate a reduction in immediate intubation‐related complications such as hypoxic or hypotensive events. However, the increased use of apnoeic oxygenation in the checklist period suggests enhanced specific procedural aspects and that the checklist serves as a valuable cognitive aid in preparing for this common yet high‐risk procedure. Further studies are warranted to investigate the checklist's effectiveness in improving patient outcomes and ensuring adherence in different healthcare settings and patient populations.

## Author Contributions

A.N., I.M.F. and J.K. planned and designed the study. U.T. and coworkers at the ICU developed the checklist based on reviewed literature. A.N., J.K., I.M.F. and A.S. analysed and interpreted the patient data and wrote the manuscript together with U.T. All authors read and approved the final manuscript.

## Ethics Statement

The study was approved by the Swedish ethical review authority (Etikprövningsmyndigheten) with the reference number Dnr 2023‐03310‐01.

Consent

The need for consent was waived as the study had a retrospective design.

## Conflicts of Interest

The authors declare no conflicts of interest.

## Data Availability

The data that support the findings of this study are available from the corresponding author upon reasonable request.

## Appendix B

**TABLE B1:** Exploratory subgroup analyses on patients intubated for respiratory failure with immediate urgency.

	Standard period (*n* = 7)	Checklist period (*n* = 8)	*p*
Primary outcome			
SpO_2_ < 90%^a^	6 (86%)	1 (14%)^a^	0.03
SAP < 90 mmHg^a^	1 (17%)^a^	4 (50%)	0.30
Secondary outcomes			
Lowest median SpO_2_ ^a^ (%)	83 (75–88)^a^	93 (88–100)	0.03
Emergency surgical airway	1 (14%)	0 (0%)	0.47
Cardiovascular collapse^b^	0 (0%)^a^	0 (0%)	
Cardiac arrest	0 (0%)	0 (0%)	
Oesophageal intubation	0 (0%)	0 (0%)	
Severe arrhythmia	0 (0%)	0 (0%)	
Death (procedural)	0 (0%)	0 (0%)	
Change of method/operator	2 (29%)	0 (0%)	0.20
Duration of intubation (s)	495 (51–1575)^c^	59 (44–106)^c^	0.26
Intubation difficulty^a^			
Easy	4 (57%)	6 (75%)	0.61
Complicated	2 (29%)	2 (25%)	1.00
Impossible	1 (14%)	0 (0%)	0.47
Number of attempts^a^			
1	5 (83%)^a^	8 (100%)	0.43
2	1 (17%)^a^	0 (0%)	0.43
30‐day mortality	3 (43%)	1 (13%)	0.28
Length of ICU stay (days)	3 (3–11)	9.5 (3–16)	0.61
Length of mechanical ventilation (days)^a^	3 (3–10)	10 (2–15)^c^	0.73
Other outcomes^a^			
SpO_2_ < 80%	3 (50%)^a^	0 (0%)	0.06
SpO_2_ < 70%	0 (0%)^a^	0 (0%)	
SAP < 80 mmHg	1 (17%)^a^	3 (38%)	0.58
SAP < 70 mmHg	1 (17%)^a^	2 (25%)	1.00
Lowest median SAP^a^ (mmHg)	110 (93–111)^c^	95 (74–107)	0.28
Intubated on ICU day			
1st day	5 (71%)	4 (50%)	0.61
2nd day	0 (0%)	0 (0%)	
3rd day	0 (0%)	2 (25%)	0.47
Other	2 (29%)	2 (25%)	1.00
Preoxygenation procedure			
Tight facial mask^a^	6 (86%)	5 (63%)	0.57
NIV^a^	0 (0%)	3 (38%)	0.20
HFNC^a^	0 (0%)	1 (13%)	1.00
Other^a^	2 (29%)	0 (0%)	0.20
Intubation procedure			
On‐call hours intubation^c^	6 (86%)	5 (63%)	0.57
Mask ventilation during preoxygenation^d^	3 (43%)	0 (0%)	0.08
Apnoeic oxygenation^a^	0 (0%)	3 (38%)	0.20
With standard nasal cannula	0 (0%)	2 (28%)	0.47
With HFNC	0 (0%)	1 (13%)	1.00
Video laryngoscopy^a^	6 (86%)^a^	7 (88%)	1.00
Intubation stylet^a^	2 (33%)^a^	7 (88%)	0.09
Operator experience > 5 years	7 (100%)	6 (75%)	0.47
Oxygen support before intubation decision	7 (100%)	8 (100%)	
Facial mask^a^	2 (29%)	2 (25%)	1.00
HFNC^a^	0 (0%)	1 (13%)	1.00
NIV^a^	2 (33%)^a^	2 (29%)^a^	1.00
Other	1 (14%)	0 (0%)	0.47
Unknown method^a^	2 (29%)	3 (38%)	1.00

*Note:* Data are number (%) or median (IQR). SpO_2_, peripheral arterial oxygen saturation; SAP, systolic arterial pressure. Lowest median SpO_2_ and SAP are from intubation up to 5 min after. Duration of intubation is defined as time from laryngoscopy to end‐tidal carbon dioxide measurement. ICU, intensive care unit; NIV, non‐invasive ventilation; HFNC, high‐flow nasal cannula. A *p* < 0.05 was considered significant.

^a^ Missing data for one patient.

^b^ Defined as SAP < 65 mmHg.

^c^ Missing data in up to three patients.

^d^ Missing data in four patients.
